# Disseminated intravascular coagulation presenting as symmetrical peripheral gangrene: a case report

**DOI:** 10.1186/s13256-019-2117-5

**Published:** 2019-07-12

**Authors:** Vineet Jain, Khan Afreen, Jyotsana Kumari, Tanveer Mir, Bilal Wani, Romit Bhushan

**Affiliations:** Department of Medicine, Hamdard Institute of Medical Sciences & Research (HIMSR), New Delhi, India

**Keywords:** Symmetrical peripheral gangrene (SPG), *P. vivax*, Malaria, DIC

## Abstract

**Introduction:**

*Plasmodium vivax* was traditionally thought to be benign; however, nowadays it presents with a myriad of systemic complications like cerebral malaria, acute respiratory distress syndrome, acute kidney injury, acute pancreatitis, hepatic dysfunction, and disseminated intravascular coagulation, which were earlier attributed only to *Plasmodium falciparum* malaria. Here we report a case of a middle-aged man who presented with disseminated intravascular coagulation manifesting as symmetrical peripheral gangrene. What makes this case more interesting is that the malaria isolated was *Plasmodium vivax* instead of *Plasmodium falciparum*. Such findings were previously reported, but this is the first case where the patient was managed conservatively with antimalarial drugs without the need for amputation, which focuses on the very important role of early diagnosis and timely management.

**Case presentation:**

A 44-year-old Indian man from north India presented with history of fever of 2 days’ duration with severely painful cold extremities. No pulse could be recorded on examination. A diagnosis of symmetrical peripheral gangrene was made. During the etiological evaluation, *Plasmodium vivax* malaria was found leading to disseminated intravascular coagulation causing this complication. He was started on artesunate and lumefantrine combination therapy and he recovered completely without the requirement of amputation.

**Conclusion:**

This case highlights the non-benign nature of *Plasmodium vivax* and its emerging complications. Also it correlates symmetrical peripheral gangrene with *Plasmodium vivax* malaria. It also emphasizes the importance of timely diagnosis and intervention to reduce mortality and morbidity.

## Introduction

Malaria is a protozoan disease transmitted by the bite of infected *Anopheles* mosquitoes. It is transmitted in 91 countries containing 3 billion people and causes approximately 1200 deaths each day [1]. Six species of the genus *Plasmodium* cause nearly all malarial infections in humans. These are *Plasmodium falciparum*, *Plasmodium vivax*, two morphologically identical sympatric species of *Plasmodium ovale*, *Plasmodium malariae*, and *Plasmodium knowlesi* [1]. The two major human malaria species in India are *P. falciparum* and *P. vivax* [2].

Symmetrical peripheral gangrene (SPG) is characterized by distal ischemic damage in two or more extremities, without large vessel obstruction. This syndrome has been reported in several conditions such as infections, disseminated intravascular coagulation (DIC), and low cardiac output states; it is rarely associated with *P. falciparum* and mixed malarial infections [3, 4].

We report a case of isolated *P. vivax* malaria presenting with DIC manifesting as SPG. This case is unusual because DIC and SPG have been reported extremely rarely in the literature in association with isolated *P. vivax* malaria [5–7]. Amputations were required in all reported cases to date, however, in the present case, our patient recovered completely with antimalarial and conservative treatment emphasizing the role of early diagnosis and initiation of treatment.

## Case presentation

A 44-year-old Indian man, resident of New Delhi, India, a known alcoholic, presented with a 2 day history of fever. His fever was 39.44 °C (103 °F), continuous and was associated with chills and rigors. Two days after onset of fever, he developed painful bluish discoloration of both hands and feet. There was no history of bluish discoloration of tongue, breathlessness, or chest pain. There was no history of hypertension, diabetes, malaria, or any chronic illness in this patient.

**Fig. 1:**
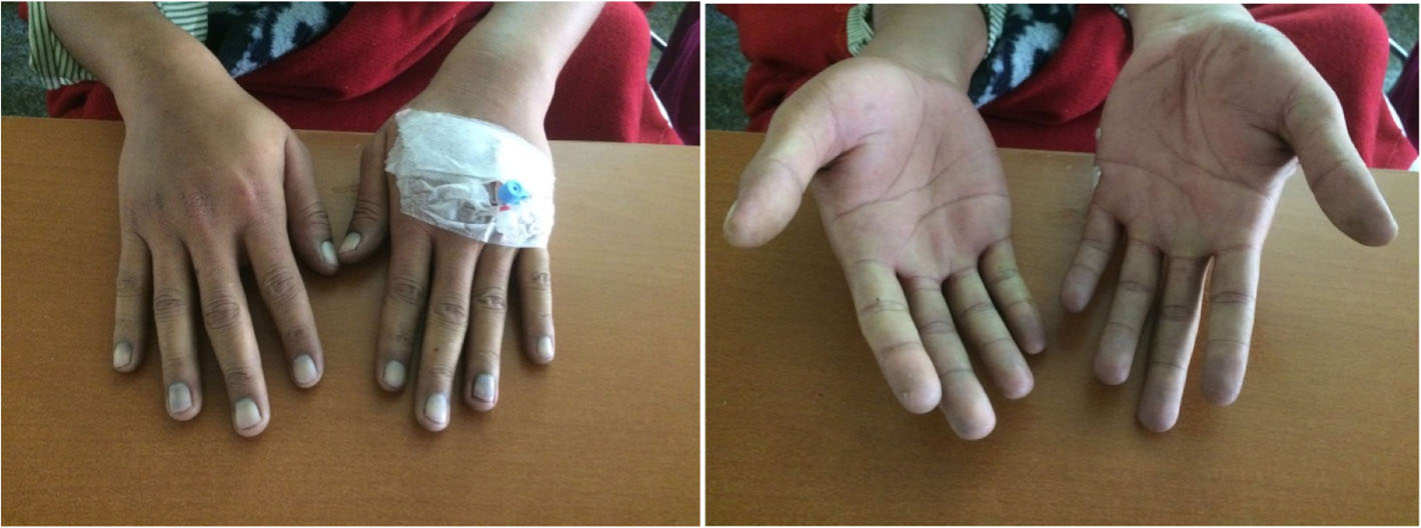
Bluish discoloration of nails and fingertips of both hands

On clinical examination he was conscious, oriented, and well hydrated. His pulse was 80 beats per minute. Bilateral radial and dorsalis pedis arteries were feeble with bluish discoloration of fingers (Fig. 1) and toes. His other peripheral pulses (carotid and femoral arteries) were palpable but low in volume. Blood pressure was not recordable in both upper limbs and was 90/60 mmHg in both his lower limbs. Both hands and feet were pale and cold to touch and there was marked tenderness in both forearm and calf muscles. There was no cyanosis of tongue or nose and there was no lymphadenopathy. The rest of the clinical examination was unremarkable.

A provisional diagnosis of symmetrical arterial ischemia/obstruction with impending gangrene was made and he was investigated further.

His hemoglobin was 15.9 g/dL (13–18 g/dL), hematocrit 44.8% (40–50%), total leukocyte count 3.9 × 10^3^/mm^3^ (4–11 × 10^3^/mm^3^), and platelet count 8000/mm^3^ (150,000–400, 000/mm^3^). Renal and liver functions were deranged with blood urea 90 mg/dL (15–45 mg/dL), serum creatinine 2.6 mg/dL (0.5–1.5 mg/dL), serum bilirubin 12.70 mg/dL (0.2–1.2 mg/dL), direct bilirubin 9.86 mg/dL (0.0–0.2 mg/dL), indirect bilirubin 2.84 mg/dL (0.0–1.0 mg/dL), albumin 2.4 g/dL (3.4–5.0 g/dL), aspartate transaminase 116 IU/L (5–40 IU/L), alanine transaminase 46 IU/L (5–40 IU/L), and alkaline phosphatase 116 IU/L (45–116 IU/L). His prothrombin time was 27.3 (control value 13); his international normalized ratio (INR) was 2.30. His random blood sugar was 64 mg/dL and arterial blood gas analysis was unremarkable.

Arterial Doppler of both upper and lower limbs was normal and did not reveal any thrombus in any of the arteries. Radiography of his chest was normal.

**Fig. 2:**
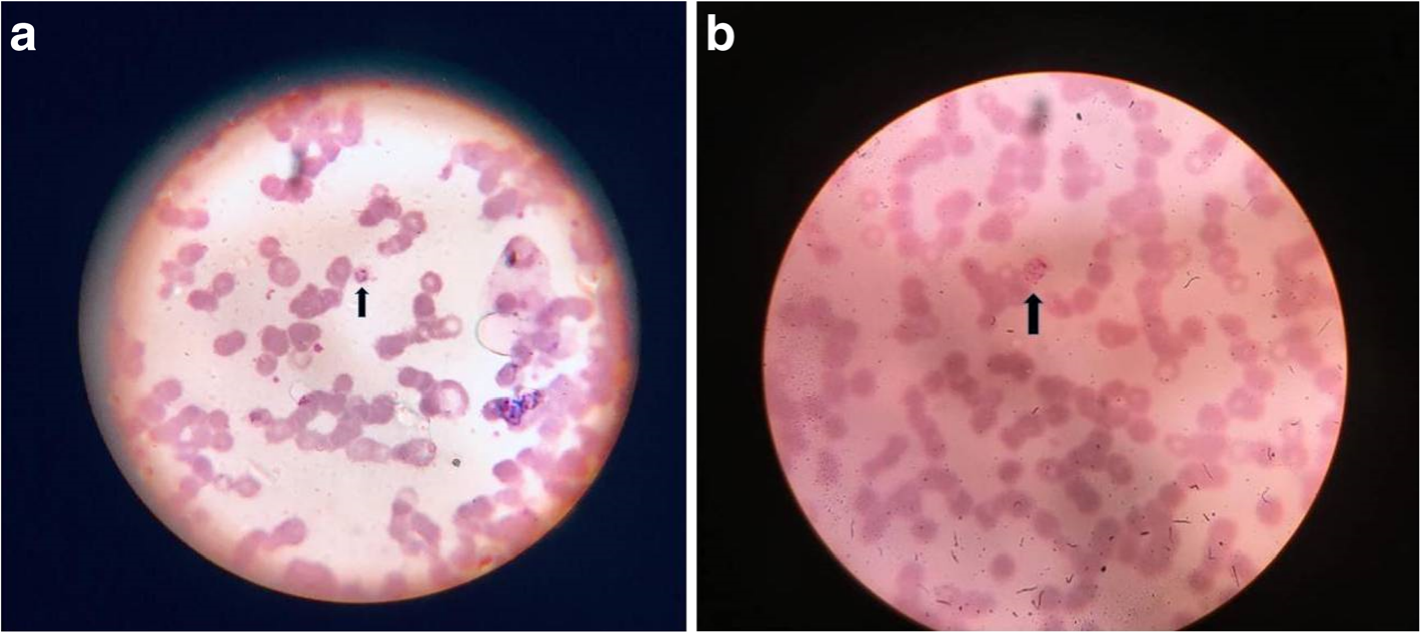
**a** Ring forms (arrow) of *Plasmodium vivax* (magnification × 1000). **b** Schizonts (arrow) of *Plasmodium vivax* (magnification × 1000)

A peripheral smear (using Giemsa stain) for malaria revealed ring forms and schizonts of *P. vivax* (Fig. 2a, b). The level of parasitemia in thick blood films was 2+ which corresponds to 11–100 parasites/100 thick films fields examined. This was later confirmed by a malaria antigen rapid diagnostic test (RDT) which detected *P. vivax* lactate dehydrogenase (LDH) ruling out a mixed infection with *P. falciparum*. NS1 antigen and dengue serology were negative. Fibrin degradation products was > 20 μg/ml (< 5). His D-dimer level was 26.52 μg/mL (< 0.5) fibrinogen-equivalent units. Tests for antinuclear antibodies and antineutrophil cytoplasmic antibodies were negative.

He was treated with injection artesunate 120 mg followed by lumefantrine 480 mg and artemether 80 mg two times per day for 3 days followed by primaquine 15 mg (0.25 mg/kg) per day for 14 days; his glucose-6-phosphate dehydrogenase (G6PD) levels were normal. He also received six units of platelet concentrates, along with cautious use of intravenously administered fluids.

The response to this line of treatment was striking and gratifying. He became afebrile by the second day of admission and by the third day all peripheral pulses were well felt along with normalization of blood pressure in all his limbs. Bluish discoloration of both hands and feet started fading by the third day and pain disappeared in the involved regions. His laboratory investigations normalized by sixth day of admission following treatment except for slightly raised bilirubin. He was discharged and no clinical or laboratory abnormality was reported on follow-ups at 2 weeks and 6 weeks.

## Discussion

*P. falciparum* causes the majority of severe and fatal malaria cases and has overshadowed the public health importance of *P. vivax* malaria.

Complications in severe malaria are either sequestration related, such as cerebral malaria, renal dysfunction, hepatic dysfunction, and acute respiratory distress syndrome (ARDS), or non-sequestration related, such as anemia and thrombocytopenia [8]. Non-sequestration-related complications are known to occur in *P. vivax* infection quite frequently [8]. *P. vivax* is less pathogenic than *P. falciparum* in otherwise healthy patients, but can cause complicated and severe disease.

SPG is a well-documented but rare clinical syndrome. It is characterized by symmetrical distal ischemic damage leading to gangrene of two or more sites in the absence of large vessel obstruction or vasculitis. DIC is considered to be the final common pathway for the pathogenesis of SPG, irrespective of the etiology. It is proposed to be a cutaneous marker of the same [9].

DIC is associated with a number of clinical conditions; generally involving activation of systemic inflammation [10]. The common underlying conditions include sepsis, trauma, malignancies, transfusion reactions, acute pancreatitis, obstetric complications, and severe toxic reactions [10].

Severe malaria has been associated with clinically apparent bleeding or DIC [11, 12]. Coagulopathy in severe malaria may result from several pathological processes such as thrombocytopenia, platelet dysfunction, coagulation activation, defects in inhibitors of coagulation, cytokine activation, endothelial cell activation, cytoadherence, and impaired fibrinolysis [11]. SPG caused by malaria is very rare, and most of the cases are associated with *P. falciparum* [3].

In a recent study, non-overt DIC was identified in more than 70% of patients of severe *P. vivax* malaria by thromboelastography as well as the conventional coagulation tests [13]. However, there have been very few reports of an association of SPG with isolated *P. vivax* malaria infection. Arora *et al.* [5] reported the index case of malaria with isolated *P. vivax* infection complicated by SPG involving bilateral hands and feet. Two other cases [6, 7] were reported of patients presenting with similar complications later. The management of SPG remains in early identification of the underlying cause and its prompt treatment along with correction of DIC if present. Amputation has been reported in all the cases reported to date. However, in the present case, our patient did not progress to develop dry gangrene and improved with antimalarial and conservative management underlining the importance of early diagnosis and treatment.

Intriguingly all case reports of SPG in *P. vivax* have been from India [5–7]. Whether there are genetic factors (host related or pathogen related) or environmental issues at play can be speculated. This would require detailed study and a larger pool of cases. One can also speculate that dehydration, a known risk factor for DIC, delay in seeking treatment, and inappropriate treatment initially by alternative practitioners may also be contributory factors.

## Conclusion

The present case highlights SPG as a rare complication of *P. vivax* malaria. It also emphasizes the need for a high index of suspicion of this clinical entity to differentiate it from conditions such as collagen vascular diseases, thrombotic thrombocytopenic purpura, and other conditions associated with peripheral gangrene. Early recognition and institution of appropriate treatment can prevent progression of gangrene and consequent requirement of amputation.

## Abbreviations

ARDS: Acute respiratory distress syndrome
DIC: Disseminated intravascular coagulation
G6PD: Glucose-6-phosphate dehydrogenase
INR: International normalized ratio
LDH: Lactate dehydrogenase
*P. falciparum*: *Plasmodium falciparum*
*P. vivax*: *Plasmodium vivax*
RDT: Rapid diagnostic test
SPG: Symmetrical peripheral gangrene
